# Supplementary material for: Rapid diagnosis of periodontitis, a feasibility study using MALDI-TOF mass spectrometry
Source: PLoS One. 2020 Mar 13;15(3):e0230334. doi: 10.1371/journal.pone.0230334 (PMC7069628; doi:10.1371/journal.pone.0230334)
Supplement: S1 Table — (PDF) [file pone.0230334.s001.pdf]

S1 Table : List of epidemiological data recording during the medical questionnaire.

Age (years)
Current smokers (subjects who smoked at least 100 cigarettes in their lifetime and who currently smoke cigarettes)
Former smokers (subjects who smoked at least 100 cigarettes in their lifetime but who had quit smoking at least 6 month prior the study)
Diabetes (HbA1c < 7)
Cardiovascular disease
Hypothyroidism (TSH<4 mUI)
Arthritis
Respiratory disease
Anti-diabetic medication[†]
Antibiotics[†]
Anti-inflammatory[†]
Antihypertensive[†]
Anticoagulants[1]
Thyroid hormone thyroxine (T4) [†]
Hormonal contraception[†]
Stress

[†] Medication within a month
