# Supplementary material for: Rapid diagnosis of periodontitis, a feasibility study using MALDI-TOF mass spectrometry
Source: PLoS One. 2020 Mar 13;15(3):e0230334. doi: 10.1371/journal.pone.0230334 (PMC7069628; doi:10.1371/journal.pone.0230334)
Supplement: S2 Table — (PDF) [file pone.0230334.s002.pdf]

mass	p (value)
2620	1,70685E-10
13461	1,46055E-07
3372	2,70977E-07
3550	2,66192E-07
6352	3,35328E-08
12692	1,05311E-07
7746	6,57802E-05
3443	1,54696E-07
6735	1,82551E-07
3519	4,66734E-07
4139	4,32182E-05
3482	2,37874E-07
7775	3,89894E-05
7855	0,023081262
4116	4,19845E-06
3587	1,54621E-06
2626	4,43082E-05
5379	1,8382E-05
4841	1,2906E-05
11078	0,000129751
2793	0,016651399
3492	3,1824E-06
3529	2,45965E-05
4426	0,000391118
13183	2,5432E-06
4166	0,000264597
3813	1,5898E-05
4965	0,000190803
3877	8,05285E-05
4903	9,97466E-07
3611	3,24185E-05
3628	0,004108815
11160	0,000116426
6027	0,000379692
2784	7,37553E-07
2519	0,000973038
3834	1,07293E-05
2896	0,01883831
4816	0,000117643
2522	0,001502418
6062	0,00012334
4438	0,000152773
4243	0,002239614
5536	0,148311488
8148	0,00246868
3708	2,01627E-05
2538	0,004199434
2744	0,001972065
5002	0,000701523

12771	9,20942E-05
2879	0,018147369
10840	0,000369643
4416	0,000634932
5947	0,001010412
3405	8,67898E-06
5588	0,00636462
3290	0,001935596
3780	0,009524251
2612	0,001012151
4475	0,014664627
3258	0,101232833
2975	0,001178658
3237	0,012048484
2411	0,004667613
3122	0,007165755
5659	0,006982299
2226	0,309604746
2760	0,002749546
4280	0,001162888
15513	0,00142822
3668	0,001298933
2692	0,049086623
6681	0,329354925
2494	0,000177172
3892	0,053812885
5726	0,211767136
4059	0,003424233
2681	0,001962694
2143	0,003570768
4374	0,026688873
4333	0,338992861
2241	0,008308979
2721	0,004162806
4777	0,367457492
8977	0,117289131
8948	0,086237169
3715	0,050277131
4531	0,142407193
2859	0,101594352
5227	0,002592561
2838	0,025591765
2821	0,081366931
3276	0,03285309
3568	0,008821409
3317	0,002754776
3322	0,041222681
3190	0,0319616
5797	0,003343999
2660	0,357458646

2582	0,000732791
6181	0,004119797
6891	0,024343576
6904	0,00619959
10444	0,010651471
9096	0,242337592
2940	0,226984554
2776	0,001238677
2041	0,034022472
2738	0,004661156
3201	0,062239159
4037	0,09307172
2080	0,000521428
6243	0,075272706
3928	0,010981046
2700	0,029988282
7157	0,35699815
2120	0,036285444
2643	0,141288289
3056	0,017693844
3034	0,00573361
3858	0,006710537
5434	0,036924205
7267	0,008203159
2919	0,005112286
2220	0,393433507
3153	0,234124184
3967	0,208992727
4067	0,081353285
2558	0,011627858
11006	0,196175536
2259	0,07465997
2085	0,003083359
3981	0,06808533
4932	0,000848444
4550	0,010875974
5469	0,092356556
2322	0,012541036
3222	0,140966097
2936	0,26823315
7451	0,017454214
2425	0,050926757
4005	0,258692127
7520	0,157240472
2952	0,240376922
5971	0,052774385
2105	0,746014397
3753	0,063407724
2888	0,036252935
3651	0,010358446

2994	0,217461331
2012	0,942332744
9060	0,226039375
5441	0,025987111
11720	0,371708095
3089	0,123438453
2337	0,258565306
4322	0,139164681
5457	0,107734449
2432	0,038711249
2451	0,073784319
2357	0,225934838
7349	0,062172934
6640	0,698280095
5504	0,132399116
5425	0,957014345
6945	0,273867523
4193	0,068923188
10920	0,048672528
2067	0,187178496
14689	0,043252841
5037	0,116425883
3207	0,187395869
5887	0,053055504
2956	0,438998196
4078	0,836860301
3333	0,929940996
5265	0,736485533
5695	0,507105776
3354	0,903751283
6325	0,175732526
5136	0,727498537
5074	0,120815647
6983	0,278896142
3015	0,994706446
5115	0,474197307
2439	0,075053871
2027	0,096147647
2180	0,479672759
4089	0,167289312
5609	0,623765607
2263	0,70794406
2281	0,525505593
4636	0,651993004
2391	0,166196251
2154	0,151124702
4723	0,990391305
4678	0,961625215
2375	0,413779095
4574	0,598044514

4873	0,330196068
2127	0,310082437
6285	0,46211656
2297	0,878576327
5864	0,443306262
5192	0,528862072
2462	0,780832462
5104	0,750423676
2164	0,815492575
4493	0,903422946
7494	0,827856985
2204	0,844596505
5154	0,673100973
3104	0,554267517
5416	0,916961856
5175	0,888392817
4664	0,60045099
4223	0,840792242
