# Supplementary material for: Rapid diagnosis of periodontitis, a feasibility study using MALDI-TOF mass spectrometry
Source: PLoS One. 2020 Mar 13;15(3):e0230334. doi: 10.1371/journal.pone.0230334 (PMC7069628; doi:10.1371/journal.pone.0230334)
Supplement: S3 Table — (PDF) [file pone.0230334.s003.pdf]

mass	p value
10586	1,95188E-09
3775	1,71608E-09
5296	1,98375E-09
4944	2,28756E-05
11359	1,00108E-07
11324	1,82456E-07
5893	1,25036E-06
4235	1,81488E-10
11447	2,64245E-08
5728	1,33232E-08
10190	1,54779E-08
2477	4,7308E-05
11317	7,23495E-07
5682	2,35869E-08
5134	0,131441962
7571	0,000966602
5661	2,27448E-07
4197	0,000219131
3613	1,68998E-07
2686	0,000136488
12762	2,54482E-06
2900	1,12194E-05
2211	8,47227E-07
12695	1,81066E-06
2483	0,005080905
2185	6,24188E-05
2970	6,28197E-07
4967	0,001828049
13335	1,90733E-05
2164	6,48006E-05
13281	6,67688E-06
2635	0,000120351
2642	4,55137E-05
13343	1,13195E-05
2229	0,000106194
4176	0,001529188
12776	7,40491E-06
4986	0,015260889
6391	0,000125637
13298	4,63851E-06
13200	8,86992E-06
4157	0,001006554
15132	0,006406948
11778	2,07695E-05
16666	0,001564782
2452	0,000276948
11039	0,004457401
6352	5,11787E-06
5071	0,009925616

5006	0,018235068
13161	1,52639E-05
16691	0,001203356
6428	0,004120668
5027	0,029594686
3655	0,000234406
5581	0,02623185
6644	0,000138393
2618	0,000402072
3931	0,000717974
16707	0,001366295
11086	0,016185005
3709	0,000209323
3672	0,001042949
3750	0,00067075
6972	0,000953426
2703	0,887857407
5425	0,002865683
3877	0,019003395
2200	0,001405764
13413	6,41943E-05
4138	0,001960785
13238	0,00032073
3373	0,003603513
3091	0,010865598
2023	0,002683822
2493	0,187359229
8570	0,002205409
2421	0,013312341
11010	0,024039011
6672	0,00188979
3732	0,003803255
13457	0,013167106
13464	0,024944573
4049	0,049994362
3631	0,00526872
6926	0,004773072
3444	0,000995903
11125	0,17126608
4329	0,02962255
2725	0,070581478
6894	0,00859893
7354	0,008828899
6586	0,004115766
13776	0,012796769
13784	0,009981637
2067	0,014219679
7349	0,005728886
11002	0,044911678
4129	0,00296053

4492	0,155300032
10449	0,004743884
4596	0,030072999
6736	0,375166774
13871	0,028865106
2145	0,027276501
2245	0,246119592
2276	0,161488012
4821	0,572729533
6280	0,31810615
2580	0,307052884
2921	0,063246418
5154	0,104725651
5160	0,531974742
3587	0,050584432
2011	0,465532247
2467	0,014299287
5048	0,384948096
10842	0,00885452
4529	0,133859242
2602	0,035357084
2359	0,092951721
2597	0,02290792
13534	0,10003089
11160	0,143357301
2054	0,555500931
6169	0,008180917
2127	0,031561283
2886	0,017023792
2943	0,031048107
2321	0,413658575
14308	0,57227314
7009	0,023202135
6775	0,862683117
2410	0,048077193
3526	0,115239337
3548	0,043103107
2334	0,332842787
2096	0,480474457
4376	0,735219353
4424	0,543253199
3331	0,036674583
3395	0,130136001
3468	0,120371255
4629	0,66576526
13549	0,29091875
13602	0,237811159
2156	0,079468073
5500	0,124201456
5509	0,054001262

2315	0,270239401
3413	0,28261076
4774	0,433786554
7939	0,534338684
10912	0,285244222
2378	0,150026235
2111	0,768285447
5099	0,24134167
3463	0,150210232
2365	0,104438964
5229	0,340639288
2830	0,555153739
6000	0,509196945
14692	0,456865264
7159	0,520161173
6187	0,900022411
3486	0,226354418
10925	0,418676474
2510	0,596469213
2289	0,337418941
5485	0,781757566
3507	0,641586675
10971	0,675840541
4288	0,903831315
3039	0,478431167
5112	0,878707917
5380	0,50424492
