# Supplementary material for: Rapid diagnosis of periodontitis, a feasibility study using MALDI-TOF mass spectrometry
Source: PLoS One. 2020 Mar 13;15(3):e0230334. doi: 10.1371/journal.pone.0230334 (PMC7069628; doi:10.1371/journal.pone.0230334)
Supplement: S4 Table — (PDF) [file pone.0230334.s004.pdf]

mass	p value
3038	3,08248E-06
2627	3,249E-07
2783	3,79922E-07
3194	3,69023E-05
4065	0,000115516
4931	0,001439576
3077	7,61848E-05
14693	3,38312E-05
2818	8,33758E-05
2407	0,000102576
11720	2,94906E-05
2498	4,67124E-05
4158	4,72944E-05
2664	0,072088021
7351	9,53321E-05
2923	0,000149242
3671	0,004911022
2068	0,000413791
2493	0,000355781
7572	0,002188329
2183	0,078544613
3444	0,017062359
4175	0,00040264
5386	0,021644334
2466	0,012539123
2203	0,005892278
7421	0,000907899
15135	0,002830118
4130	0,0022316
14306	0,005302144
3776	0,000143669
2126	0,933120207
4775	0,0053882
7158	0,003958762
13784	0,01637436
5047	0,007976121
2161	0,089391417
2700	0,005830362
2299	0,022321757
2268	0,391168299
2586	0,334879994
5228	0,007806064
3615	0,001522026
3589	0,029517784
5297	0,082606253
4138	0,015885422
4431	0,186024811
5947	0,093525849
11364	0,07605655

10922	0,010882448
3373	0,07930436
7227	0,078574947
2427	0,012068769
2011	0,047661795
4848	0,042853507
13469	0,017272416
3543	0,032103247
2728	0,025348405
3631	0,021755302
2338	0,985925921
3486	0,421882008
4546	0,02617319
2240	0,257573969
10846	0,073730455
4675	0,032030349
13196	0,133380089
3520	0,087368334
2090	0,033304585
2351	0,134744699
6384	0,036966338
4495	0,023871099
6895	0,085198155
2110	0,957097843
4236	0,102209034
6937	0,067551278
4904	0,018474398
3877	0,626284206
14010	0,172677861
2026	0,552348578
3655	0,007188343
3748	0,297767437
5731	0,286293576
4596	0,752214486
5426	0,436949164
3331	0,095084563
2742	0,37099677
11323	0,276422458
4969	0,166538145
2484	0,27020167
13236	0,306080072
11002	0,099495763
4120	0,214809453
5501	0,234304113
10448	0,166164518
7008	0,700513583
6739	0,120038468
6979	0,21300525
6354	0,805626079
6431	0,937816276

6751	0,168795343
12776	0,706193134
12700	0,37966212
5864	0,093734395
3731	0,879102338
3558	0,230675772
6189	0,406161318
3917	0,895110539
6588	0,681447848
3710	0,587841535
10482	0,731099603
5683	0,714383757
4819	0,699857793
5268	0,579105686
2391	0,101049515
3406	0,637279967
6644	0,169950105
5004	0,182193124
5186	0,688649798
5661	0,890500906
4985	0,701331492
13278	0,91805821
10590	0,913384406
6821	0,933383674
3391	0,767108165
