# Supplementary material for: Rapid diagnosis of periodontitis, a feasibility study using MALDI-TOF mass spectrometry
Source: PLoS One. 2020 Mar 13;15(3):e0230334. doi: 10.1371/journal.pone.0230334 (PMC7069628; doi:10.1371/journal.pone.0230334)
Supplement: Attachment — Submitted filename: Response to Reviewers.pdf [file pone.0230334.s005.pdf]

Professor MONNET-CORTI Virginie, DDS-Ph.D
Head Department of Periodontology
Chairman of the post-graduate programs in Periodontology and Periodontal Plastic Surgery
IRD *6236*, IFR48. MEPHI et VITROME- IHU Méditerranée Infection-
Aix-Marseille Université 27 Boulevard Jean Moulin, 13385 Marseille France
https://orcid.org/0000-0002-3642-6981
virginie.corti@univ-amu.fr

January 12, 2020

To: Tommaso Lomonaco, Ph.D

Academic Editor

PLOS ONE

Dear Editor,

We would like to thank you and the Reviewers for having taken in reviewing our manuscript so thoroughly. We apologize for not being explicit enough and we fully understand your comments and those of the reviewers. We have carefully taken all the remarks into consideration in preparing our revised manuscript. Our point-by-point responses to the comments are outlined below. We also propose another title for our manuscript, “Rapid diagnosis of periodontitis, a feasibility study using MALDI-TOF mass spectrometry”.

Sincerely,

Professor MONNET-CORTI Virginie, DDS-Ph.D

Response to Reviewer #1:

We thank you for your review of our manuscript. We have carefully taken into consideration your comments and answered each of your points below.

<u>Major comments:</u>

1) How were proteins extracted form each sample? This experimental part in the materials and methods section is lacking. Saliva is an aqueous fluid containing high amount of salts, cells and debris that should be removed in order to investigate the proteomic content. Similarly, is not clear how the proteins were extracted from GCF and subgingival dental plaque samples.

Answer: We corrected the paragraph "Mass spectrometry" (line 175) in the manuscript in order to better detail our methodology. Proteins were not extracted and samples were directly spotted on steel target and analysed by MALDI-TOS MS. We used the conventional technique described for bacterial identification called profiling (or fingerprinting) which is based on the comparison of spectra of unknown bacteria to database containing spectra of known reference bacteria. In this approach, intact cells can directly be mixed with MALDI matrix (Sandrin TR et al. Mass Spectrom Rev. 2013). Therefore, we chose to use whole samples to generate spectra that were compared to each other in order to assess if MALDI-TOF profiling applied to saliva, gingival crevicular fluid and dental plaque could be used as a diagnostic tool in periodontology.

2) From the mass spectrometry paragraph in the materials and methods section is stated that each sample was spotted onto the MALDI target with a volume of 0.5 uL. Was the total protein concentration of each sample evaluated? In order to compare the proteomic profiles of the two groups (Parodontitis and healthy subjects) the amount of proteins from each sample (saliva, GCF and subgingival dental plaque) spotted onto the MALDI target has to be the same. Before mass spectrometry analysis the total protein amount has to be estimated in each sample in order to spot onto the target the same amount of proteins (ug) from each sample. There are different protein assays available on the market to evaluate the total protein concentration. My concern, is that the differences in the proteomic profiles, observed by the authors, could be indeed affected by the differences in the protein amount of each samples and not related with the disease status of the subjects included in the study.

Answer: We used MALDI-TOF MS to perform profiling of saliva, gingival crevicular fluid and dental plaque from periodontitis and healthy subjects, which is not a quantitative analysis. (Sandrin TR et al, Mass Spectrom Rev. 2013). This approach was performed in our laboratory for arthropods identification (El Hamzaoui B et al, PLoS Negl Trop Dis. 2018; Tandina F et al, Parasitology. 2018).

3) Line 155: “In addition, the usual negative and positive controls were employed for each target”. What is the usual negative and positive control? Please clarify this.

Answer: We clarified this in the paragraph “Mass spectrometry” (line 175). The calibration of the mass spectrometer is performed with a commercial solution (BTS: Bacterial standard test) (line 190). In addition, in each MALDI target, 4 spots were made with an inactivated strain of Escherichia coli as the positive control (objective score > 2, 1) and 4 spots were made with matrix solution alone as the negative control (objective score <1, 5) (lines 192-194).

4) Although low resolving power mass spectrometers are used, it should be standard practice to mention external mass spectrometer calibration accuracy.

Answer: External mass spectrometer calibration accuracy was +/-300ppm.

5) How was the mass spectra pre-processing performed (i.e. mass spectra normalization, peak picking)?

Answer: We precised the mass spectra pre-processing in the paragraph “Mass spectrometry” (line 175). Quality criteria of the spectrum for global aspect and intensity were checked: intensity above 10^4 arbitrary units (AU), horizontal baseline curve and presence of visually identifiable peaks (lines 195-197).

<u>Minor comments</u>:

1) Line 24 – please explain the abbreviation GCF and add: gingival crevicular fluid (GCF).

Answer: We clarified it.

2) Line 39 – Change “in” with “of”: “peptide profiles in saliva, GCF and dental plaque”.

Answer: We changed it.

3) Line 351 – add the word “first”: “In the present study, we developed, for the…time […]”.

Answer: We added it.

4) Lines 394 and 397 – Please remove the “a” and “b” superscript.

Answer: We removed it.

5) Please check the order of the Reference List. The first paper cited in the Introduction, Papapanou et al, 2018 correspond to the reference number 20 in the Reference list. Please order the cited papers in order of appearance in the text.

Answer: We modified the order of the reference list.

Response to Reviewer #2

We thank you for your review of our manuscript. We totally agreed with all your comments and answered each of your points below.

Major comments:

1) The paper is lacking information about the assays for protein concentration of saliva, GCF and dental plaque and consequently, any kind of normalization for protein concentration has not been performed. The total amount of biological samples was not determined and without this important information it is uncertain whether the statistical difference among disease groups and healthy controls is due to a real difference in protein expression levels or purely reflects different amount of collected samples. These are the fundamental evaluations in order to standardize the protocol and to proceed with MS analysis for comparative studies. The authors should start from this stage to make a quantitative analysis comparable.

Answer: The aim of this pilot study was not to identify biomarker but to assess the feasibility and diagnostic contribution of profiling using MALDI-TOF applied to saliva, GCF and dental plaque from periodontitis and healthy subjects. We clarified it in the introduction of our manuscript. Profiling was described as a conventional approach for bacterial identification by comparing spectra of unknown bacteria to libraries that contain spectra of known reference bacteria. In this technique, intact cells can directly be mixed with MALDI matrix (Sandrin TR et al, Mass Spectrom Rev. 2013). We choose to adapt this non quantitative approach to oral fluids and dental plaque samples to generate spectra that were compared to each other and to evaluate if they could classify subjects according to their periodontal status. This approach has already been performed in our laboratory for arthropods identification (El Hamzaoui B et al, PLoS Negl Trop Dis. 2018; Tandina F et al, Parasitology. 2018).

2) Another important step in the standardization protocol is the storage conditions adopted for the biological samples. The authors wrote that: “Samples were immediately stored at 4°C and analyzed within 24 to 48 hours”. A number of studies demonstrated that the proteins can undergo degradation processes and that collection/handling and storage conditions may influence the stability of endogenous peptidome of biological fluids (Del Boccio et al, Ann Neurol. 2007; del Campo et al., Biomark Med. 2012; Preianò el al., Proteomics. 2016) therefore the authors should have preliminary performed experiments to identify the storage conditions which enable optimal preservation of biological specimen in order to ensure that the variation of protein expression levels in spectra reflects real biological differences rather than experimental artefacts. In this respect, the claims are NOT properly placed in the context of the previous literature.

Answer: We thank you for this pertinent remark and we argued our choice of methodology in the discussion. Del Boccio et al. (2007) and Del Campo et al. (2012) analysed cerebrospinal fluid among different storage conditions. As we did not use the same samples, we couldn’t use the results of these two studies, even though Del Campo et al. (2012) “recommended to store the samples at 4°C, since it can be easily done and it does not modify the biochemical results”. Preianò et al. (2016) found that best preserved signatures were obtained when GCF samples were stored at -80°C for 1 month compared to samples stored at -20°C during 3 months but the authors did not evaluate storage at 4°C and shorter durations. As Bellagambi et al. (Microchem J, 2018) and Lomonaco et al. (PLoS One, 2014; Microchem. J, 2018) found, oral fluids storage at 4°C during 4 weeks can preserve substances like cortisol, uric acid or WAR, RR/SS-warfarin and RS/SR-warfarin alcohols. Despite the lack of data available concerning saliva, GCF and dental plaque storage conditions before direct MALDI-TOF MS analysis, the same conditions applied to all of our samples thus ensuring the correct comparability of our results.

3) The authors should provide more information about MALDI sample preparation protocol, instruments settings adopted for spectra acquisition, and about the parameters for data processing (such as signal to noise and spectra normalization). The authors should investigate the impact of more strictly analytical variables on the generation of reliable MALDI-TOF spectra. It is well known that before starting MALDI based protein profiling study, it is necessary to assess the reliability of profiles with exploratory experiments in order to increase the analytical performances and the robustness of the results; in particular, they should have preliminary analyzed the influence of MALDI sample preparation by modulating the matrix composition and the analyte/matrix ratio in order to optimize the reproducibility of the MALDI peptidome profiles.

Answer: Our study was a pilot study and we clarified it in the introduction of our manuscript. The aim was to assess the feasibility and diagnostic contribution of protein profiling using MALDI-TOF applied to saliva, GCF and dental plaque from periodontitis and healthy subjects. We hypothesized that rapid routine and blinded MALDI-TOF analysis could accurately classify these three types of samples according to the periodontal state. We chose a methodology following as closely as possible the conventional MALDI-TOF

protocol (used in routine diagnostic, i.e Microflex LT, Brucker and a mass range of 2-20 kDa) and we did not look for biomarkers identification.

4) The authors have not shown how reproducible the system is. They have not indicated the number of replicate analyses and the resulting coefficient of variations (CVs) considering that for diagnostic test CVs must fall in a range of 1.5-10% (Albrethsen J. Reproducibility in protein profiling by MALDI-TOF mass spectrometry. Clin Chem 2007; 53:852–8).

Answer: The number of replicate analyses was 6 (line 176 and line 184). The spectra generated for each sample of each subject were merged in order to develop a main spectra (MSP) (lines 202-205).

5) The authors must provide more information about the parameters for data processing (such as signal to noise and spectra normalization) and criteria of statistical analysis. Additionally, it must be considered that for quantitative analysis, it is necessary to use an internal standard, for example an endogenous control protein that can be observed within the same MS run for all the sample analysis. Alternatively, at least spectra normalization is required.

Answer: We provided more informations about the parameters for date processing in paragraphs "Mass spectrometry" (line 175) and "Bioinformatics analysis" (line 199). The signal-to-noise applied was = 3 (line 204). The calibration of the MS was fully automated and performed with a commercial solution (BTS: Bacterial standard test) and the procedure was completely automatic (BiotyperRTC user manual) (lines 189-192).

6) The authors wrote: "All the patients were selected based upon periodontal status, regardless of the other criteria (e.g., systemic disease or disease/infection that may affect the periodontal health status, use of antibiotics or immunosuppressant medication within 3 months, current or former smokers), in order to reduce selection bias". Are the investigators sure that the above mentioned conditions, such as use of antibiotics or anti-inflammatory do not interfere with the analysis of the molecular profile precluding the eligibility of the subjects for the present study?

Answer: The aim of our pilot study was to assess the feasibility and diagnostic contribution of profiling using MALDI-TOF applied to saliva, GCF and dental plaque from periodontitis and healthy subjects. This idea has already been investigated by Tand et al. (Clin Chim Acta. 2019) using saliva, GCF and serum samples in a very limited population, free of periodontal risk factors. In such conditions, their results deviated from target population and could not be applied to a routine large-scale diagnostic test. That's why we decided to randomly choose a large healthy and periodontitis population in order to be closer to the clinical reality and to assess whether there were co-factors that could impact on the diagnosis.

7) Why did the authors decide to consider only the top 10 ranking peaks for the discriminant analysis? I think that this choice could lead to a loss of information.

Answer: We have chosen the 10 most discriminating peaks because only 7 were needed to build the diagnostic decision tree for saliva and only 9 for GCF and dental plaque. The objective was to select the minimum number of peaks to obtain the best sensitivity and specificity (lines 271-272).

Minor comments:

• Figure 1 shows spectra obtained from saliva, GCF and dental plaque samples. Do these spectra derive from representative individuals? In order to make data clearer to the reader, the spectra should be shown in the appropriate m/z range for the best detection of molecular features of samples. Moreover, the top 10 ranking peaks found differentially expressed between the periodontitis and the control groups for each type of sample should be highlighted in the figure. Considering that the study focuses on MALDI-MS analysis, the choice of the representative spectra is very important. Are the authors sure that the selected spectra in Fig 1 are the most representative? Particularly, for GCF, the spectra appears poorly resolved and few peaks are characterized by a good S/N ratio.

Answer : You are right, we have chosen an example that we are not sure is representative. We removed the figure.

• Figure 3 should be also revised, in order to make it clearer.

Answer : We totally agree with your remark and we removed the figure.

• The figure legends do not have all the information required to easily follow the discussion, so the authors must provide more details to the reader.

Answer : We removed figure 2 and figure 3 that were not clear.

• In the discussion, experimental data are not appropriately commented and some link between literature studies and authors findings should be revised. The authors have not treated the literature fairly.

Answer : You are right and we clarified our manuscript.

Response to Academic Editor, Tommaso Lomonaco, Ph.D

We thank you for considering our manuscript and for allowing us the opportunity to respond to the peer review comments. We found all the comments very helpful to improve our manuscript. We thank you for the six articles that you suggested and we added them, as you advised us, in the introduction and in the discussion. We hope now that the revised manuscript will be suitable for publication.
