# Supplementary material for: Rapid diagnosis of periodontitis, a feasibility study using MALDI-TOF mass spectrometry
Source: PLoS One. 2020 Mar 13;15(3):e0230334. doi: 10.1371/journal.pone.0230334 (PMC7069628; doi:10.1371/journal.pone.0230334)
Supplement: Attachment — Submitted filename: Response to Reviewers.docx [file pone.0230334.s006.docx]

**Professor MONNET-CORTI Virginie,** DDS-Ph.D

Head Department of Periodontology

Chairman of the post-graduate programs in Periodontology and Periodontal Plastic Surgery

IRD *6236*, IFR48. MEPHI et VITROME- IHU Méditerranée Infection-

Aix-Marseille Université 27 Boulevard Jean Moulin, 13385 Marseille France

https://orcid.org/0000-0002-3642-6981

[virginie.corti@univ-amu.fr](mailto:virginie.corti@univ-amu.fr)

February 25, 2020

To: Tommaso Lomonaco, Ph.D

Academic Editor

PLOS ONE

Dear Editor,

We would like to thank you and the Reviewers for your comments on our manuscript. We have edited our manuscript and our point-by-point responses to the comments are outlined below.

Sincerely,

Professor MONNET-CORTI Virginie, DDS-Ph.D

**Response to Academic Editor, Tommaso Lomonaco, Ph.D**

We thank you for your comments on the manuscript and have edited the manuscript to address their concerns.

We hope now that the revised manuscript will be suitable for publication.

1. I suggest to move the table 1 in the supplementary information.

**Answer :** We changed it.

2. Please explain at which variable the p-value was related (table 2).

**Answer :** We clarified the title. The table 2 shows the demographic characteristics contrasting the subjects from the periodontitis group and control group, with the p-value resulting from the between-group comparison of all subjects.

3. Please explain the meaning of the percentage in the brackets (table 3).

**Answer :** We clarified the title. The percentage in each box refers to the relative frequency in relation to the total of 67 periodontitis subjects.

4. Please explain the meaning of the number in the brackets (L374).

**Answer :** We clarified the title of the table corresponding. The sentence “A large proportion of our periodontitis group was classified into stages III (46,3%) and IV (41,8%)” means that 46,3% of periodontitis subjects are classified in a stage III and 41,8% in a stage IV according to the new classification of periodontal diseases (Chicago, 2017).

5. Please include the RSD regarding replicate analysis on the same sample.

**Answer :** We added it (line 199) and modified the paragraph “bioinformatics analysis” (line 192) to complete the methodology.

In response to the reviewers' comments, we clarified that we did not identify proteins and did not attempt to evaluate the proteins differentially expressed between the two groups. We evaluated the differentially expressed peaks and compared the spectra of the different subjects according to the fingerprinting principle. We have completed the details of the spectra analysis (lines 195-201) and specified the interest of the MALDI-TOF MS technique in the comparison of spectra (lines 389-393).

**Response to Reviewer #1:**

We thank you for your comment on our manuscript.

The paper was not properly improved and I can not suggest its publication. The experimental design is not technically sound. In order to determine differentially expressed proteins (as stated even in the abstract, lines 33-34) he authors should have determined the total protein concentration of each sample and to spot the same amount of proteins, as a consequence all of the results are not consistent.

**Answer :** As stated in our abstract, we determined the differentially expressed peaks and not the differentially expressed proteins. We carried out a pattern matching of spectra profiles coming from different groups (periodontitis and control group). We did not identify proteins. We provide more information in the discussion (lines 389-393). The spectra comparison is based on protein fingerprinting. As Maldi-TOF MS is semiquantitative (Sandrin et al., 2012; Albrethsen, 2007), it allows to work on the relative frequency of peaks within the same spectrum, the comparison of these relative frequencies between spectra being ensured by normalization (TIC, total ion current) (Meuleman et al., 2008; Borgaonkar et al., 2010; Sandrin et al., 2012). This mechanism is the basis of the Maldi-tof bacterial identification technique currently used in routine (Rupf et al., 2005; Sandrin et al., 2008; Freiwald & Sauer, 2009).

**Response to Reviewer #2:**

We thank you for your comment on our manuscript.

In the revised version of the manuscript PONE-D-19-31089R1 entitled: “Rapid diagnosis of periodontitis, a feasibility study using MALDI-TOF mass spectrometry” the Authors have improved the overall quality of the study however the manuscript still remains scientifically and methodologically not accurate because of

1) lack of experiments concerning the short-term storage at 4° of saliva, GCF and dental plaque. For comparative profiling studies, storage time must be the same for all the samples analyzed: the protein profile of a sample stored for 48 h at 4° may not be compared to that stored for 24 h at 4°. Storage time must be standardized to ensure the comparability of the results.

**Answer :** We argued our choice of methodology in the discussion. Preianò et al. (2016) found that best preserved signatures were obtained when GCF samples were stored at -80°C for 1 month compared to samples stored at -20°C during 3 months but the authors did not evaluate storage at 4°C and shorter durations. As Bellagambi et al. (Microchem J, 2018) and Lomonaco et al. (PLoS One, 2014; Microchem. J, 2018) found, oral fluids storage at 4°C during 4 weeks can preserve substances like cortisol, uric acid or WAR, RR/SS-warfarin and RS/SR-warfarin alcohols. However, we agree that further studies would be necessary to assess the optimal time frame for a routine diagnostic test and added it in the discussion.

2) lack of precise quantification (for example the lack in the use of internal standards for MALDI-TOF analysis; moreover none of the various kinds of normalization for protein concentration has been performed), especially for peptide and protein profile obtained by MALDI-TOF MS, might be very dangerous because MALDI-TOF is not inherently quantitative.

These limitations could affect the results of the statistical analysis and raise doubts whether the statistical difference among disease groups and healthy controls is due to a real difference in protein expression levels or purely reflects different amounts of collected samples.

Therefore this reviewer strongly suggests that the limitations of this study should be highlighted and openly and critically discussed by the Authors.

All these issues are much more imperative in a “feasibility” study as the Authors claim in the new proposed title of the revised manuscript “Rapid diagnosis of periodontitis, a feasibility study using MALDI-TOF mass spectrometry” It would be more correct, for the benefit of the readers, to replace the new proposed title “Rapid diagnosis of periodontitis, a feasibility study using MALDI-TOF mass spectrometry”

with a new one for example “Rapid diagnosis of periodontitis, a feasibility study using routine and blinded MALDI-TOF analysis”. In the opinion of this reviewer it is better to clarify the non-conventional use of MALDI-TOF mass spectrometry in this study starting from the title.

Considering that the data presented here are very weak because not well supported by the requested experiments, systematic bias in the design of this study may cause erroneous results causing false expectations in the wide readership of PLos ONE. Nevertheless, in a reasonable scenario it could be also possible that such study could lead to controversial debate on a possible misuse of MALDI as already happened for SELDI pioneering studies.

**Answer :** We provided more information in the discussion. The spectra comparison is based on protein fingerprinting. As Maldi-TOF MS is semiquantitative (Sandrin et al., 2012; Albrethsen, 2007), it allows to work on the relative frequency of peaks within the same spectrum, the comparison of these relative frequencies between spectra being ensured by normalization (TIC, total ion current) (Meuleman et al., 2008; Borgaonkar et al., 2010; Sandrin et al., 2012). This mechanism is the basis of the Maldi-tof bacterial identification technique currently used in routine (Rupf et al., 2005; Sandrin et al., 2008; Freiwald & Sauer, 2009) (lines 389-393).
